# Supplementary material for: Analysis on the control of the black tiger shrimp in the America from the perspective of international cooperation
Source: PLoS One. 2024 May 31;19(5):e0300833. doi: 10.1371/journal.pone.0300833 (PMC11142530; doi:10.1371/journal.pone.0300833)
Supplement: S1 File — (DOCX) [file pone.0300833.s001.docx]

**Appendix 1**

Take the derivatives of *G_B_*_1_ with respect to (13), and take the derivatives of *G_B_*_2_ with respect to (14), and set them equal to zero, we can get:

$G_{B1}^{\text{*}}\left(t\right)=\frac{b_{1}+\frac{\partial V_{B1}}{\partial x_{B1}}}{c_{1}}$ (43)

$G_{B2}^{\text{*}}\left(t\right)\text{=}\frac{b_{2}+b_{2}\ln\left(\text{1+}h_{B}\right)+\frac{\partial V_{B2}}{\partial x_{B2}}\left(f_{2}-f_{B}\right)}{c_{2}+c_{B}}$ (44)

Substituting (43) into (13) and substituting (44) into (14), we can get:

$\rho V_{B1}=b_{1}\frac{b_{1}+\frac{\partial V_{B1}}{\partial x_{B1}}}{c_{1}}-\frac{c_{1}}{2}\left(\frac{b_{1}+\frac{\partial V_{B1}}{\partial x_{B1}}}{c_{1}}\right)^{2}+lx_{B1}\left(t\right)+\frac{\partial V_{B1}}{\partial x_{B1}}\left[f_{1}\frac{b_{1}+\frac{\partial V_{B1}}{\partial x_{B1}}}{c_{1}}-\delta x_{B1}\left(t\right)\right]$ (45)

$\begin{matrix} \rho V_{B2}=b_{2}\frac{b_{2}+b_{2}\ln\left(\text{1+}h_{B}\right)+\frac{\partial V_{B2}}{\partial x_{B2}}\left(f_{2}-f_{B}\right)}{c_{2}+c_{B}}+b_{2}\ln\left(\text{1+}h_{B}\right)\frac{b_{2}+b_{2}\ln\left(\text{1+}h_{B}\right)+\frac{\partial V_{B2}}{\partial x_{B2}}\left(f_{2}-f_{B}\right)}{c_{2}+c_{B}} \\ -\frac{c_{2}+c_{B}}{2}\left[\frac{b_{2}+b_{2}\ln\left(\text{1+}h_{B}\right)+\frac{\partial V_{B2}}{\partial x_{B2}}\left(f_{2}-f_{B}\right)}{c_{2}+c_{B}}\right]^{2}+lx_{B2}\left(t\right) \\ +\frac{\partial V_{B2}}{\partial x_{B2}}\left[\left(f_{2}-f_{B}\right)\frac{b_{2}+b_{2}\ln\left(\text{1+}h_{B}\right)+\frac{\partial V_{B2}}{\partial x_{B2}}\left(f_{2}-f_{B}\right)}{c_{2}+c_{B}}-\delta x_{B2}\left(t\right)\right] \end{matrix}$ (46)

Let $V_{B1}^{*}=k_{1}x_{B1}+k_{2}$, $V_{B2}^{\text{*}}=k_{3}x_{B2}+k_{4}$, wherein, *k*_1_, *k*_2_, *k*_3_ and *k*_4_ are all constants. The parameters of the optimal social welfare function can be obtained by calculation as follows:

$\left\{\begin{matrix} k_{1}=\frac{l}{\rho+\delta} \\ k_{2}=\frac{1}{\rho}b_{1}\frac{b_{1}+\frac{l}{\rho+\delta}}{c_{1}}-\frac{c_{1}}{2}\frac{1}{\rho}\left(\frac{b_{1}+\frac{l}{\rho+\delta}}{c_{1}}\right)^{2}+\frac{l}{\rho+\delta}\frac{1}{\rho}f_{1}\frac{b_{1}+\frac{l}{\rho+\delta}}{c_{1}} \end{matrix}\right.$ (47)

$\left\{\begin{matrix} k_{3}=\frac{l}{\rho+\delta} \\ k_{4}=\frac{1}{\rho}b_{2}\frac{b_{2}+b_{2}\ln\left(\text{1+}h_{B}\right)+\frac{l}{\rho+\delta}\left(f_{2}-f_{B}\right)}{c_{2}+c_{B}}+\frac{1}{\rho}b_{2}\ln\left(\text{1+}h_{B}\right)\frac{b_{2}+b_{2}\ln\left(\text{1+}h_{B}\right)+\frac{l}{\rho+\delta}\left(f_{2}-f_{B}\right)}{c_{2}+c_{B}} \\ -\frac{c_{2}+c_{B}}{2}\frac{1}{\rho}\left[\frac{b_{2}+b_{2}\ln\left(\text{1+}h_{B}\right)+\frac{l}{\rho+\delta}\left(f_{2}-f_{B}\right)}{c_{2}+c_{B}}\right]^{2} \\ +\frac{l}{\rho+\delta}\frac{1}{\rho}\left(f_{2}-f_{B}\right)\frac{b_{2}+b_{2}\ln\left(\text{1+}h_{B}\right)+\frac{l}{\rho+\delta}\left(f_{2}-f_{B}\right)}{c_{2}+c_{B}} \end{matrix}\right.$(48)

Therefore, it can be concluded that:

$V_{B1}^{*}=\frac{l}{\rho+\delta}x_{B1}+\frac{1}{\rho}b_{1}\frac{b_{1}+\frac{l}{\rho+\delta}}{c_{1}}-\frac{c_{1}}{2}\frac{1}{\rho}\left(\frac{b_{1}+\frac{l}{\rho+\delta}}{c_{1}}\right)^{2}+\frac{l}{\rho+\delta}\frac{1}{\rho}f_{1}\frac{b_{1}+\frac{l}{\rho+\delta}}{c_{1}}$ (49)

$\begin{matrix} V_{B2}^{\text{*}}=\frac{1}{\rho}b_{2}\frac{b_{2}+b_{2}\ln\left(\text{1+}h_{B}\right)+\frac{l}{\rho+\delta}\left(f_{2}-f_{B}\right)}{c_{2}+c_{B}}+\frac{1}{\rho}b_{2}\ln\left(\text{1+}h_{B}\right)\frac{b_{2}+b_{2}\ln\left(\text{1+}h_{B}\right)+\frac{l}{\rho+\delta}\left(f_{2}-f_{B}\right)}{c_{2}+c_{B}} \\ -\frac{c_{2}+c_{B}}{2}\frac{1}{\rho}\left[\frac{b_{2}+b_{2}\ln\left(\text{1+}h_{B}\right)+\frac{l}{\rho+\delta}\left(f_{2}-f_{B}\right)}{c_{2}+c_{B}}\right]^{2}+\frac{l}{\rho+\delta}x_{B2} \\ +\frac{l}{\rho+\delta}\frac{1}{\rho}\left(f_{2}-f_{B}\right)\frac{b_{2}+b_{2}\ln\left(\text{1+}h_{B}\right)+\frac{l}{\rho+\delta}\left(f_{2}-f_{B}\right)}{c_{2}+c_{B}} \end{matrix}$(50)

In this case,

$G_{B1}^{\text{*}}\left(t\right)=\frac{b_{1}+\frac{l}{\rho+\delta}}{c_{1}}$ (51)

$G_{B2}^{\text{*}}\left(t\right)\text{=}\frac{b_{2}+b_{2}\ln\left(\text{1+}h_{B}\right)+\frac{l}{\rho+\delta}\left(f_{2}-f_{B}\right)}{c_{2}+c_{B}}$ (52)
