# Supplementary material for: Analysis on the control of the black tiger shrimp in the America from the perspective of international cooperation
Source: PLoS One. 2024 May 31;19(5):e0300833. doi: 10.1371/journal.pone.0300833 (PMC11142530; doi:10.1371/journal.pone.0300833)
Supplement: S2 File — (DOCX) [file pone.0300833.s002.docx]

**Appendix 2**

Take the derivatives of *G_M_*_1_ with respect to (15), and take the derivatives of *G_M_*_2_ with respect to (16), and set them equal to zero, we can get:

$G_{M1}^{\text{*}}\left(t\right)=\frac{b_{1}+q_{M1}+\frac{\partial V_{M1}}{\partial x_{M1}}f_{1}}{c_{1}+c_{M1}}$ (53)

$G_{M2}^{*}\left(t\right)=\frac{q_{M2}+f_{2}\frac{\partial V_{M2}}{\partial x_{M2}}}{c_{M2}}$ (54)

Substituting (53) into (15) and substituting (54) into (16), we can get:

$\begin{matrix} \rho V_{M1}=b_{1}\frac{b_{1}+q_{M1}+\frac{\partial V_{M1}}{\partial x_{M1}}f_{1}}{c_{1}+c_{M1}}-\frac{\left(c_{1}+c_{M1}\right)}{2}\left(\frac{b_{1}+q_{M1}+\frac{\partial V_{M1}}{\partial x_{M1}}f_{1}}{c_{1}+c_{M1}}\right)^{2}+q_{M1}\frac{b_{1}+q_{M1}+\frac{\partial V_{M1}}{\partial x_{M1}}f_{1}}{c_{1}+c_{M1}} \\ +lx_{M1}\left(t\right)+\frac{\partial V_{M1}}{\partial x_{M1}}\left[f_{1}\frac{b_{1}+q_{M1}+\frac{\partial V_{M1}}{\partial x_{M1}}f_{1}}{c_{1}+c_{M1}}-\delta x_{M1}\left(t\right)\right] \end{matrix}$ (55)

$\begin{matrix} \rho V_{M2}=q_{M2}\frac{q_{M2}+f_{2}\frac{\partial V_{M2}}{\partial x_{M2}}}{c_{M2}}-\frac{c_{M2}}{2}\left(\frac{q_{M2}+f_{2}\frac{\partial V_{M2}}{\partial x_{M2}}}{c_{M2}}\right)^{2}+lx_{M2}\left(t\right) \\ +\frac{\partial V_{M2}}{\partial x_{M2}}\left[f_{2}\frac{q_{M2}+f_{2}\frac{\partial V_{M2}}{\partial x_{M2}}}{c_{M2}}-\delta x_{M2}\left(t\right)\right] \end{matrix}$ (56)

Let $V_{M1}^{*}=k_{5}x_{M1}+k_{6}$,$V_{M2}^{\text{*}}=k_{7}x_{M2}+k_{8}$, wherein, *k*_5_, *k*_6_, *k*_7_ and *k*_8_ are all constants. The parameters of the optimal social welfare function can be obtained by calculation as follows:

$\left\{\begin{matrix} k_{5}=\frac{l}{\rho+\delta} \\ k_{6}=\frac{1}{\rho}b_{1}\frac{b_{1}+q_{M1}+\frac{l}{\rho+\delta}f_{1}}{c_{1}+c_{M1}}-\frac{1}{\rho}\frac{\left(c_{1}+c_{M1}\right)}{2}\left(\frac{b_{1}+q_{M1}+\frac{l}{\rho+\delta}f_{1}}{c_{1}+c_{M1}}\right)^{2}+\frac{1}{\rho}q_{M1}\frac{b_{1}+q_{M1}+\frac{l}{\rho+\delta}f_{1}}{c_{1}+c_{M1}} \\ +\frac{1}{\rho}\frac{l}{\rho+\delta}f_{1}\frac{b_{1}+q_{M1}+\frac{l}{\rho+\delta}f_{1}}{c_{1}+c_{M1}} \end{matrix}\right.$ (57)

$\left\{\begin{matrix} k_{7}=\frac{l}{\rho+\delta} \\ k_{8}=\frac{1}{\rho}q_{M2}\frac{q_{M2}+f_{2}\frac{l}{\rho+\delta}}{c_{M2}}-\frac{c_{M2}}{2}\frac{1}{\rho}\left(\frac{q_{M2}+f_{2}\frac{l}{\rho+\delta}}{c_{M2}}\right)^{2}+\frac{l}{\rho+\delta}\frac{1}{\rho}f_{2}\frac{q_{M2}+f_{2}\frac{l}{\rho+\delta}}{c_{M2}} \end{matrix}\right.$ (58)

Therefore, it can be concluded that:

$\begin{matrix} V_{M1}^{*}=\frac{l}{\rho+\delta}x_{M1}+\frac{1}{\rho}b_{1}\frac{b_{1}+q_{M1}+\frac{l}{\rho+\delta}f_{1}}{c_{1}+c_{M1}}-\frac{1}{\rho}\frac{\left(c_{1}+c_{M1}\right)}{2}\left(\frac{b_{1}+q_{M1}+\frac{l}{\rho+\delta}f_{1}}{c_{1}+c_{M1}}\right)^{2} \\ +\frac{1}{\rho}q_{M1}\frac{b_{1}+q_{M1}+\frac{l}{\rho+\delta}f_{1}}{c_{1}+c_{M1}}+\frac{1}{\rho}\frac{l}{\rho+\delta}f_{1}\frac{b_{1}+q_{M1}+\frac{l}{\rho+\delta}f_{1}}{c_{1}+c_{M1}} \end{matrix}$ (59)

$\begin{matrix} V_{M2}^{\text{*}}=\frac{l}{\rho+\delta}x_{M2}+\frac{1}{\rho}q_{M2}\frac{q_{M2}+f_{2}\frac{l}{\rho+\delta}}{c_{M2}}-\frac{c_{M2}}{2}\frac{1}{\rho}\left(\frac{q_{M2}+f_{2}\frac{l}{\rho+\delta}}{c_{M2}}\right)^{2} \\ +\frac{l}{\rho+\delta}\frac{1}{\rho}f_{2}\frac{q_{M2}+f_{2}\frac{l}{\rho+\delta}}{c_{M2}} \end{matrix}$ (60)

In this case,

$G_{M1}^{\text{*}}\left(t\right)=\frac{b_{1}+q_{M1}+\frac{l}{\rho+\delta}f_{1}}{c_{1}+c_{M1}}$ (61)

$G_{M2}^{*}\left(t\right)=\frac{q_{M2}+f_{2}\frac{l}{\rho+\delta}}{c_{M2}}$ (62)
