# Supplementary material for: Analysis on the control of the black tiger shrimp in the America from the perspective of international cooperation
Source: PLoS One. 2024 May 31;19(5):e0300833. doi: 10.1371/journal.pone.0300833 (PMC11142530; doi:10.1371/journal.pone.0300833)
Supplement: S3 File — (DOCX) [file pone.0300833.s003.docx]

**Appendix 3**

Take the derivatives of *G_E_*_1_ with respect to (17), and take the derivatives of *G_E_*_2_ with respect to (18), and set them equal to zero, we can get:

$G_{E1}^{\text{*}}\left(t\right)=\frac{b_{1}+q_{E1}+\frac{\partial V_{E1}}{\partial x_{E1}}f_{1}}{c_{1}}$ (63)

$G_{E2}^{\text{*}}\left(t\right)=\frac{q_{E2}+\frac{\partial V_{E2}}{\partial x_{E2}}\left(f_{E}+f_{2}\right)}{c_{T}}$ (64)

Substituting (63) into (17) and substituting (64) into (18), we can get:

$\begin{matrix} \rho V_{E1}=b_{1}\frac{b_{1}+q_{E1}+\frac{\partial V_{E1}}{\partial x_{E1}}f_{1}}{c_{1}}-\frac{c_{1}}{2}\left[\frac{b_{1}+q_{E1}+\frac{\partial V_{E1}}{\partial x_{E1}}f_{1}}{c_{1}}\right]^{2}+q_{E1}\frac{b_{1}+q_{E1}+\frac{\partial V_{E1}}{\partial x_{E1}}f_{1}}{c_{1}} \\ +lx_{E1}\left(t\right)+\frac{\partial V_{E1}}{\partial x_{E1}}\left[f_{1}\frac{b_{1}+q_{E1}+\frac{\partial V_{E1}}{\partial x_{E1}}f_{1}}{c_{1}}-\delta x_{E1}\left(t\right)\right] \end{matrix}$ (65)

$\begin{matrix} \rho V_{E2}=q_{E2}\frac{q_{E2}+\frac{\partial V_{E2}}{\partial x_{E2}}\left(f_{E}+f_{2}\right)}{c_{T}}-\frac{c_{T}}{2}\left[\frac{q_{E2}+\frac{\partial V_{E2}}{\partial x_{E2}}\left(f_{E}+f_{2}\right)}{c_{T}}\right]^{2}+lx_{E2}\left(t\right) \\ +\frac{\partial V_{E2}}{\partial x_{E2}}\left[\left(f_{E}+f_{2}\right)\frac{q_{E2}+\frac{\partial V_{E2}}{\partial x_{E2}}\left(f_{E}+f_{2}\right)}{c_{T}}-\delta x_{E2}\left(t\right)\right] \end{matrix}$ (66)

Let $V_{E1}^{*}=k_{9}x_{E1}+k_{10}$,$V_{E2}^{\text{*}}=k_{11}x_{E2}+k_{12}$, wherein, *k*_9_, *k*_10_, *k*_11_ and *k*_12_ are all constants. The parameters of the optimal social welfare function can be obtained by calculation as follows:

$\left\{\begin{matrix} k_{9}=\frac{l}{\rho+\delta} \\ k_{10}=\frac{1}{\rho}b_{1}\frac{b_{1}+q_{E1}+\frac{l}{\rho+\delta}f_{1}}{c_{1}}-\frac{c_{1}}{2}\frac{1}{\rho}\left[\frac{b_{1}+q_{E1}+\frac{l}{\rho+\delta}f_{1}}{c_{1}}\right]^{2}+q_{E1}\frac{1}{\rho}\frac{b_{1}+q_{E1}+\frac{l}{\rho+\delta}f_{1}}{c_{1}} \\ +\frac{l}{\rho+\delta}\frac{1}{\rho}f_{1}\frac{b_{1}+q_{E1}+\frac{l}{\rho+\delta}f_{1}}{c_{1}} \end{matrix}\right.$(67)$\left\{\begin{matrix} k_{11}=\frac{l}{\rho+\delta} \\ k_{12}=\frac{1}{\rho}q_{E2}\frac{q_{E2}+\frac{l}{\rho+\delta}\left(f_{E}+f_{2}\right)}{c_{T}}-\frac{c_{T}}{2}\frac{1}{\rho}\left[\frac{q_{E2}+\frac{l}{\rho+\delta}\left(f_{E}+f_{2}\right)}{c_{T}}\right]^{2} \\ +\frac{l}{\rho+\delta}\frac{1}{\rho}\left(f_{E}+f_{2}\right)\frac{q_{E2}+\frac{l}{\rho+\delta}\left(f_{E}+f_{2}\right)}{c_{T}} \end{matrix}\right.$ (68)

Therefore, it can be concluded that:

$\begin{matrix} V_{E1}^{*}=\frac{l}{\rho+\delta}x_{E1}+\frac{1}{\rho}b_{1}\frac{b_{1}+q_{E1}+\frac{l}{\rho+\delta}f_{1}}{c_{1}}-\frac{c_{1}}{2}\frac{1}{\rho}\left[\frac{b_{1}+q_{E1}+\frac{l}{\rho+\delta}f_{1}}{c_{1}}\right]^{2}+q_{E1}\frac{1}{\rho}\frac{b_{1}+q_{E1}+\frac{l}{\rho+\delta}f_{1}}{c_{1}} \\ +\frac{l}{\rho+\delta}\frac{1}{\rho}f_{1}\frac{b_{1}+q_{E1}+\frac{l}{\rho+\delta}f_{1}}{c_{1}} \end{matrix}$(69)$\begin{matrix} V_{E2}^{\text{*}}=\frac{l}{\rho+\delta}x_{E2}+\frac{1}{\rho}q_{E2}\frac{q_{E2}+\frac{l}{\rho+\delta}\left(f_{E}+f_{2}\right)}{c_{T}}-\frac{c_{T}}{2}\frac{1}{\rho}\left[\frac{q_{E2}+\frac{l}{\rho+\delta}\left(f_{E}+f_{2}\right)}{c_{T}}\right]^{2} \\ +\frac{l}{\rho+\delta}\frac{1}{\rho}\left(f_{E}+f_{2}\right)\frac{q_{E2}+\frac{l}{\rho+\delta}\left(f_{E}+f_{2}\right)}{c_{T}} \end{matrix}$ (70)

In this case,

$G_{E1}^{\text{*}}\left(t\right)=\frac{b_{1}+q_{E1}+\frac{l}{\rho+\delta}f_{1}}{c_{1}}$ (71)

$G_{E2}^{\text{*}}\left(t\right)=\frac{q_{E2}+\frac{l}{\rho+\delta}\left(f_{E}+f_{2}\right)}{c_{T}}$ (72)
